# ASSERT: A Blockchain-Based Architectural Approach for Engineering Secure Self-Adaptive IoT Systems

**DOI:** 10.3390/s22186842

**Published:** 2022-09-09

**Authors:** Fahed Alkhabbas, Mohammed Alsadi, Sadi Alawadi, Feras M. Awaysheh, Victor R. Kebande, Mahyar T. Moghaddam

**Affiliations:** 1 Internet of Things and People Research Center, Malmö University, 21119 Malmö, Sweden; 2 Department of Computer Science and Media Technology, Malmö University, 21119 Malmö, Sweden; 3 Department of Computer Science, Norwegian University of Science and Technology, 7491 Trondheim, Norway; 4 Department of Information Technology, Uppsala University, 75105 Uppsala, Sweden; 5 Center for Applied Intelligent Systems Research, School of Information Technology, Halmstad University, 30118 Halmstad, Sweden; 6 Institute of Computer Science, Delta Research Centre, University of Tartu, 51009 Tartu, Estonia; 7 Department of Computer Science (DIDA), Blekinge Institute of Technology, 37179 Karlskrona, Sweden; 8 The Maersk Mc-Kinney Moller Institute (MMMI), University of Southern Denmark, 5230 Odense, Denmark

**Keywords:** Internet of Things, software architecture, self-adaptive and goal-driven systems, blockchain, security, multi-agent systems

## Abstract

Internet of Things (IoT) systems are complex systems that can manage mission-critical, costly operations or the collection, storage, and processing of sensitive data. Therefore, security represents a primary concern that should be considered when engineering IoT systems. Additionally, several challenges need to be addressed, including the following ones. IoT systems’ environments are dynamic and uncertain. For instance, IoT devices can be mobile or might run out of batteries, so they can become suddenly unavailable. To cope with such environments, IoT systems can be engineered as goal-driven and self-adaptive systems. A goal-driven IoT system is composed of a dynamic set of IoT devices and services that temporarily connect and cooperate to achieve a specific goal. Several approaches have been proposed to engineer goal-driven and self-adaptive IoT systems. However, none of the existing approaches enable goal-driven IoT systems to automatically detect security threats and autonomously adapt to mitigate them. Toward bridging these gaps, this paper proposes a distributed architectural Approach for engineering goal-driven IoT Systems that can autonomously SElf-adapt to secuRity Threats in their environments (ASSERT). ASSERT exploits techniques and adopts notions, such as agents, federated learning, feedback loops, and blockchain, for maintaining the systems’ security and enhancing the trustworthiness of the adaptations they perform. The results of the experiments that we conducted to validate the approach’s feasibility show that it performs and scales well when detecting security threats, performing autonomous security adaptations to mitigate the threats and enabling systems’ constituents to learn about security threats in their environments collaboratively.

## 1. Introduction

The Internet of Things (IoT) and Artificial Intelligence (AI) technologies have enabled novel types of systems—referred to as Intelligent IoT systems—in several application domains, such as surveillance, building automation, transportation and logistics, and healthcare [1,2]. Specifically, the IoT has enabled a huge number of objects and devices, such as sensors, actuators, vehicles, and appliances, to connect and communicate, whereas the integration of AI techniques into the IoT systems makes them intelligent and autonomous [3].

Intelligent IoT systems have become more and more intertwined in almost every aspect of our daily lives. These systems can manage costly or mission-critical operations, such as managing a district’s heating or controlling autonomous vehicles, respectively. Additionally, they collect and process data that might be private or sensitive (e.g., data from our homes or patients’ health parameters). Accordingly, compromising the security of such systems has severe consequences and affects users’ trust in them. Therefore, security should be considered a primary concern when engineering intelligent IoT systems. Indeed, practitioners have specified security as a main non-functional requirement that they often consider when engineering IoT systems [4]. Likewise, in the literature, security has been recognized as the most studied quality characteristic of IoT systems [5].

The environments of IoT systems are dynamic and uncertain. For example, due to the mobility of IoT devices, it is not always feasible to foresee the devices available in users’ environments. Moreover, IoT devices are often resource-constrained with respect to processing power and energy [5]. Consequently, they can become suddenly unavailable. Likewise, they can be discovered and join IoT systems at runtime. To cope with such dynamic and uncertain environments, IoT systems can be engineered as goal-driven and self-adaptive systems. A goal-driven IoT system is composed of a dynamic set of things that connect and cooperate temporarily to achieve a goal [6].

Several approaches have been proposed to engineer goal-driven and self-adaptive IoT systems by exploiting different techniques and notions, including multi-agent systems [7,8], the web of things [9,10], feedback loops [6,8], service-oriented architectures [11,12], a microservices architecture [13], AI planning [6,14], model-driven engineering [15], and business processes [16]. However, the majority of the existing approaches focus mostly on the functional requirements and do not consider security as a main concern [17]. Moreover, most of the existing approaches developed to engineer secure self-adaptive systems adopt centralized architectures and thus are not suitable for the distributed and dynamic IoT environments [18].

Toward bridging this gap, in this paper, we propose *ASSERT*—an Approach for engineering distributed goal-driven IoT Systems that can autonomously SElf-adapt to secuRity Threats in their environments. *ASSERT*also enables the systems’ constituents to learn about security threats in their environments collaboratively. To the best of our knowledge, *ASSERT*is the first approach that takes the software engineering perspective to address the aforementioned challenges. For this purpose, *ASSERT*exploits techniques and adopts notions such as blockchain, belief–desire–intention (BDI) agents, federated learning, and feedback loops. *ASSERT*is composed of architecture, processes, and a prototype developed and used to run experiments to validate the feasibility of our approach.

The remainder of this paper is organized as follows. Section 2 presents a brief background on IoT security and the main technologies used to develop *ASSERT*. Section 3 introduces the research methodology applied to devise our *ASSERT*. Section 4 introduces *ASSERT*. Section 5 presents the experiments conducted to validate the feasibility of our approach. Section 6 discusses the approach. Section 7 surveys the related work. Finally, Section 8 concludes the paper and outlines future work directions.

## 2. Background

**IoT Security.** The security of IoT systems is of utter importance as the failure can be severe. The IoT application stack has multi-layers, including (1) the perception layer, where physical devices capture data about their surrounding environments; (2) the network layer, where the data captured by IoT devices are transmitted through the network; and (3) the application layer, where the data are processed and presented to end-users via interfaces. The majority of IoT devices and objects have limited processing and storage capabilities [5]; thus, the security mechanisms applied to traditional software systems are not applicable. Additionally, the existence of vulnerabilities in connected IoT environments further extends the threat and attack landscape [19]. Therefore, IoT systems, services, and networks are vital assets to be protected from potential attacks, starting from the design phase [20].

Attacks on IoT systems can be classified into four categories [21]: (1) *physical attacks*, which target the physical devices; (2) *network attacks*, which are generally launched remotely to perform unauthorized actions on the digital assets within an IoT network, aiming at damaging the systems; (3) *software attacks*, which strike in the form of malicious pieces of computer code and applications that can harm the IoT systems; and (4) *data attacks*, which are illegal attempts to access, harm, and steal IoT data. In this context, attack detection [22] and a continuous security evaluation are critical for IoT system sustainability [23].

**Blockchain.** Blockchain technology has come to the scene after the introduction of Bitcoin in 2009 [24]. Blockchain is a list of blocks chained together using cryptographic hash functions. Each block acts as a container holding entries called *transactions*, representing the transfer of digital assets between participants. The list keeps growing subsequently as new transactions are generated. Blockchain has a distributed architecture with several attractive features, such as data integrity, immutability, and transparency. These features have enhanced the potential of blockchain technology to be exploited beyond crypto-currencies in various fields, including supply chain management [25], electronic health record management [26], electronic voting [27], access controls [28], and the IoT. The distributed architecture of blockchain eliminates the need for a central authority to manage the network content. This opens up new opportunities for creating new frameworks beneficial to societies. For example, Blockchain has a vital role in renewable energy distribution [29], which helps to effectively manage them mainly in small grids. Further, it creates a win–win model through which end-users can generate and trade their own energy. *Consensus* is the mechanism used in blockchain to ensure all participants (nodes) within the network have an identical copy of the content. In addition to the aforementioned blockchain features, most current blockchain platforms have introduced the concept of smart contracts to develop and run applications securely on top of the blockchain in a distributed manner. A *smart contract* is a self-executing program that can automatically control, execute, and enforce agreements over the blockchain network.

**Multi-Agent IoT Systems.** An *agent* is an autonomous computer system situated in a possibly dynamic and uncertain environment [30,31]. Multi-agent systems (MAS) are distributed systems constituted by agents that interact and collaborate to achieve common goals [32]. This makes the MAS appropriate to be exploited to realize goal-driven and self-adaptive IoT systems. Indeed, there has been an increasing interest in leveraging agents’ technology to develop IoT systems [33]. However, further efforts are required to engineer self-adaptive MAS in a goal-directed manner [17,34].

A well-known model for realizing intelligent agents is the belief–desire–intention (BDI) model proposed by Rao and Georgeff [35]. BDI-agents make rational decisions and determine their actions by continuously reasoning about their (1) beliefs about themselves and their environments; (2) desires (i.e., goals); and (3) intentions (i.e., the goals that the agents commit to achieving). Our approach exploits BDI agents to realize goal-driven and self-adaptive IoT systems because BDI agents are goal-directed, autonomous, context-aware, and can communicate and collaborate to realize distributed MAS-based IoT systems.

Existing approaches developed to realize goal-driven MAS exploit other technologies and notions, including rule-based programming [36], ensembles [37,38], and interaction-oriented programming [39,40]. To summarize, exploiting agents’ technology to develop goal-driven and self-adaptive IoT systems that can operate in dynamic and uncertain environments contributes to a promising unexplored research direction in both the IoT and MAS [17,33,41].

**Self-Adaptive IoT Systems.** Self-adaptation methods equip software systems with capabilities to cope with environmental and contextual changes occurring at runtime [42,43]. Several technologies and notions can be exploited to engineer self-adaptive systems, including control theory [44], rules [45], optimization algorithms [46], machine learning [47], processes [48], and events [49]. Most existing approaches rely on predicting the uncertainties an IoT system could face during its operation. Such predictions, which are made often at design time, enable the system to make adaptations at runtime. Several configurations are generally modeled based on dynamic goals and situations. In this way, software architectures could be designed based on expectations and further adapted based on runtime dynamics [50]. Other approaches generate adaptation strategies on the fly at runtime. Predefined strategies require design effort but impose a low computation overhead, whereas runtime model generations could explore optimal solutions but demand more computation power.

One of the well-known techniques exploited to develop self-adaptive systems is the Monitor–Analyze–Plan–Knowledge (MAPE-K) control loops. In this technique, IoT systems continuously collect data (e.g., using sensors or cameras) about themselves and their environments (M). Then, the collected data are analyzed by computational components to detect changes in the systems or their environments (A). When changes are detected, plans are generated or selected to maintain the systems’ goals (P), and the plans are then executed (E). Finally, the derived knowledge is stored in the systems’ knowledge bases (e.g., for learning purposes) (K).

Finally, unlike our approach, none of the existing approaches enable dynamically formed and distributed IoT systems to automatically detect security threats and perform trustworthy adaptations to mitigate them.

**Federated Learning.** Traditional machine learning (ML) approaches require data to be collected and aggregated offline on centralized servers or Cloud nodes, where the models are trained and deployed. Such approaches are not suitable to detect security threats in distributed and dynamic IoT environments because training and deploying ML models in central nodes incur communication latency, consume the communication bandwidth, and represent single points of failure [51,52]. Distributed learning approaches are applied to allow for a more optimal utilization of resources across the Edge–Cloud continuum. However, they require users’ data to be released to the (distributed) servers. Thus, such approaches do not address the privacy and security concerns [53,54].

To overcome the aforementioned limitations, Google proposed federated learning (FL), an emerging paradigm that enables users or organizations to jointly train a machine learning model without releasing their private data [51,55,56]. Briefly, in FL settings, distributed machines collaboratively learn by training models locally, aggregating the trained models’ parameters weights and propagating the aggregated weights to all the machines.

## 3. Research Methodology

**Figure 1:**
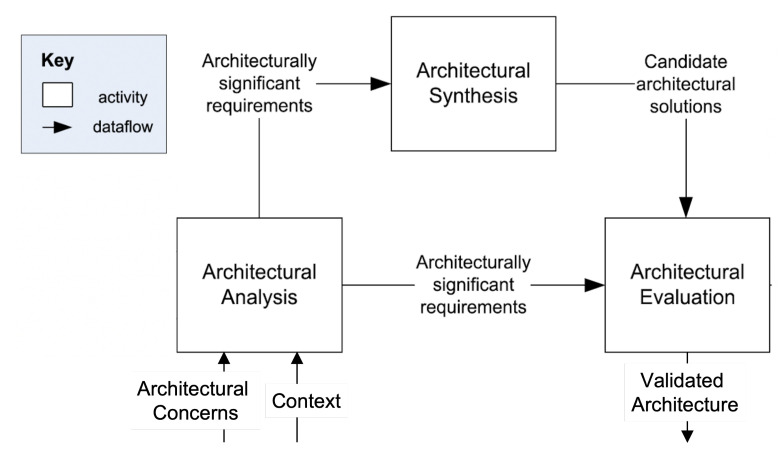
The architecture design activities [57].

To design the architecture of our approach, the general model proposed by [57] is applied. The authors devised the model by analyzing and comparing five models for engineering software architectures in practice. The model comprises three main activities, which are presented below and shown in Figure 1.

1. **Architectural Analysis:** In this activity, the problem that the approach should solve is identified. The main output of this activity is a set of Architecturally Significant Requirements (ASRs). To formulate the ASRs, the relevant architectural concerns and the context of goal-driven and self-adaptive IoT systems were analyzed [58]. According to the IEEE 1417, *architectural concerns* are interests relevant to a system’s development or operations or important to the system’s stakeholders [59]. The *context of a goal-driven and self-adaptive IoT system* concerns the settings and circumstances that have effects on the system, such as the purpose of developing the system, the current state of the technology, and the system’s development and operations processes [58,59]. The main contextual dimensions of goal-driven and self-adaptive IoT systems were depicted, together with a set of relevant scenarios and architectural concerns. Then, the set of ASRs were formulated accordingly. The results of this activity are reported in Section 4.1.
2. **Architectural Synthesis:** In this activity, a candidate architectural solution that meets the formulated ASRs was identified. First, the literature for architectures proposed to realize goal-driven and self-adaptive IoT systems was surveyed. Then, the identified architectures considering the contextual dimensions, the ASRs, and the scenarios developed during the architectural analysis activity were analyzed. Thereafter, an architecture that overcomes the shortcomings of the existing ones and meets the ASRs was proposed. The results of this activity are reported in Section 4.2.
3. **Architectural Evaluation:** In this activity, the candidate architectural solution was evaluated with respect to the ASRs. For this purpose, a prototype was developed and used to run experiments to validate the feasibility of our approach. The results of this activity are described in Section 5.

## 4. ASSERT: A Blockchain-Based Architectural Approach for Engineering Secure Self-Adaptive IoT Systems

This section introduces our approach by presenting the output of the research activities defined in Section 3.

### 4.1. Architectural Analysis

**Context.** The developmental and operational circumstances of the existing approaches developed to engineer goal-driven and self-adaptive IoT systems are summarized below. Moreover, a set of scenarios that highlight the shortcomings of these approaches is presented. The main context-related dimensions are as follows:

**C1:** Most approaches proposed to engineer self-adaptive and goal-driven IoT systems that operate in dynamic and uncertain environments do not consider security as a primary concern [17].

**C2:** Most approaches developed to engineer secure self-adaptive systems adopt centralized architectures [18]. Consequently, such architectures represent single points of failure and are not convenient for distributed IoT environments.

**Scenarios.** The smart surveillance system scenario, which builds upon the scenario presented in [60], is presented below. Then, a set of scenarios of attacks that can be conducted on the system are also presented.

The smart surveillance system is a goal-driven and self-adaptive system that aims at detecting intruders trying to enter factory buildings. The system is composed of a dynamic set of drones that patrol the area, sensors that detect suspicious behaviors, and smart cameras that move and analyze the activities happening in the vicinity of the buildings. The number of deployed drones can differ based on the size of the area they guard, their battery levels, and their connection status [61]. When an intruder is detected, the drones and cameras take photos of the intruder, issue verbal warnings, and alert the security team members about the event via their smartphones.

Below, a set of attack scenarios and responses are presented. Each scenario is described using the template proposed [62]. Specifically, we describe (1) the *actor* (e.g., a human or non-human user or the system itself) that generates a stimulus; (2) the *stimulus*, which corresponds to an event generated by the actor or the environment; (3) the *artifact*, which is (part of) the system responsible for handling the stimulus; (4) the *response*, which represents the result of managing the stimulus; and (5) the *response measure*, which determines if the response is acceptable (i.e., whether the requirement is satisfied).

SC1 *Actor*: An attacker. *Stimulus*: The attacker manages to upload and execute a malicious software on some drones to blur the videos they capture or to know the current security teams’ locations. *Artifact*: The software managing the devices and the servers running the smart surveillance system. *Response*: The devices detect the attack and reboot, non-compromised drones are instructed to replace the compromised ones, and the guards are notified (e.g., via their smartphones). *Response Measure:* The compromised devices do not lead to compromising the system or preventing it from achieving its goals.
SC2 *Actor*: An attacker. *Stimulus*: The attacker manages to upload and execute a malicious software code on one of the servers running the smart surveillance system. *Artifact*: The smart surveillance system. *Response*: The attack on the server is detected, the server is rebooted, and another server is automatically assigned the tasks of the compromised server. For that purpose, the secure server uses the context analyzed by the compromised server before being compromised. *Response Measure:* The attack on one server does not compromise the entire system and does not affect its functionalities significantly.
SC3 *Actor*: A number of attackers. *Stimulus*: The attackers perform distributed denial-of-service (DDoS) attacks on some of the servers running the smart surveillance system. *Artifact*: The smart surveillance system. *Response*: The DDoS attacks on the servers are automatically detected, the servers allow the communications only among themselves, and new servers are automatically deployed and assigned the tasks of the non-responding servers. *Response Measure:* The attack on the servers does not prevent the system from providing its services.
SC4 *Actor*: An unauthenticated drone. *Stimulus*: The drone tries to contact and send the wrong information to a server hosting part of the smart surveillance system, aiming at facilitating the penetration of the factory. *Artifact*: The server that receives the communication request sent by the fake drone. *Response*: The server detects that the drone is not authenticated, requests authentication, temporarily blocks the communication channels with the drone if it does not respond within a specific time period, and notifies other servers about the IP address of the suspected drone. *Response Measure:* The communication request initiated by the drone is rejected and the data it sent are neglected.
SC5 *Actor*: A connected device (e.g., a drone) or a server hosting (part of) the smart surveillance system. *Stimulus*: A strange behavior of the actor is detected; however, no attack is detected automatically. *Artifact*: The smart surveillance system. *Response*: The expert is notified, and they manage to detect a new type of security threat after checking the device or the server. The expert adds the newly recognized attack to the compromised entity’s knowledge base and defines a process that will be executed automatically when the attack is detected. *Response Measure:* The other system constituents are able to detect the attack and trigger the adaptation process automatically.

**Architectural Concerns.** Several studies have presented functional requirements that goal-driven and self-adaptive IoT systems should meet [9,17]. Moreover, they have developed several approaches to allow goal-driven and self-adaptive systems to meet those requirements. Specifically, they have proposed approaches to support users to achieve their goals seamlessly in arbitrary environments by enabling the dynamic formation, deployment, adaptation, and enactment of goal-driven and self-adaptive IoT systems [17]. Additionally, some proposed approaches recommend services to users based on the specific context and to configure users’ environments automatically based on their preferences. The reader is referred to [17] for more details about the existing approaches.

The Architecturally Significant Requirements (ASRs) considered when engineering *ASSERT*are presented below. Basically, security is considered as a primary concern at this stage of the work. Moreover, performance and scalability are also considered in the experiments because security measures often affect them. Other important concerns, such as privacy, reliability, and usability, will be addressed in our future work.

R1 Goal-driven and self-adaptive IoT systems should be able to detect and handle security threats automatically and autonomously.
R2 Goal-driven and self-adaptive IoT systems should collaboratively learn about security threats and use the gained knowledge to detect them automatically.
R3 Goal-driven and self-adaptive IoT systems should monitor, recognize, and evaluate the strange behaviors of their constituents.
R4 Goal-driven and self-adaptive IoT systems should be responsive when performing an increasing number of security adaptations.

### 4.2. Architectural Synthesis

To design the architecture of *ASSERT*, the architectures of the existing approaches developed to engineer goal-driven and/or self-adaptive IoT systems were analyzed, considering the scenarios and ASRs specified in Section 4.1. Most architectures are found to be centralized, and none meet the defined ASRs. The most relevant approach was proposed by [8]. The approach enables the distributed realization of autonomous goal-driven and self-adaptive systems by exploiting agents’ technology and feedback loops. However, the authors did not present the architecture of their approach and did not consider security as a primary concern. Therefore, the approach presented in [8] is refined to meet the ASRs presented in Section 4.1. Our approach supports goal-driven and self-adaptive IoT systems to cope with security threats after the systems have been dynamically formed. (See Section 4.2.1 for more details about the dynamic formation of goal-driven systems.)

**Figure 2:**
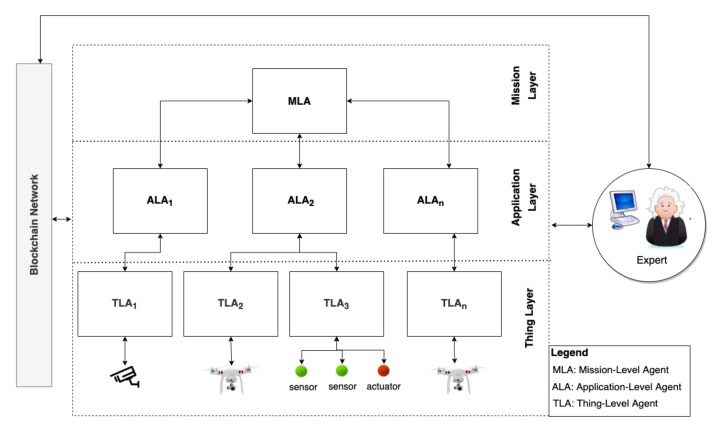
The abstract architecture of our approach.

Figure 2 shows the abstract hierarchical architecture of *ASSERT*. The architecture comprises three layers: the mission layer, application layer, and thing layer. Additionally, the architecture integrates a blockchain network, as further clarified below. *ASSERT*enables automatic security adaptations in response to (1) malicious code-injection attacks; (2) fake node attacks; and (3) (distributed) denial-of-service attacks. The framework will be extended to handle additional threats in future work.

**Mission Layer.** This layer comprises one agent, the mission-level agent (MLA), whose goal is to fulfil missions (e.g., to surveil a factory) through orchestrating multiple application-level agents. The MLA is involved in realizing all goal-driven and self-adaptive IoT systems.

**Application Layer.** This layer comprises the application-level agents (ALAs) that, following the instructions of the MLA, collaborate to achieve a mission. An ALA is an application-dependent agent whose goal is to achieve parts of the mission (e.g., to surveil a building) through orchestrating one or more thing-level agents.

**Thing Layer.** This layer comprises the thing-level agents (TLAs) that manage the actual IoT devices and objects and instruct them to perform concrete tasks (e.g., stream video and detect motion) to achieve the goals of the ALAs and, consequently, the mission.

**Blockchain Network.** This layer compromises the blockchain network used for providing an immutable and trusted single source of truth for the application and mission Layers. Additionally, blockchain is used as a distributed registry of information needed to perform trustworthy security adaptations in response to security threats. A smart contract is used for authenticating agents and securing data sharing between them.

#### 4.2.1. The Dynamic Formation and Adaptation of Goal-Driven IoT Systems

**Figure 3:**
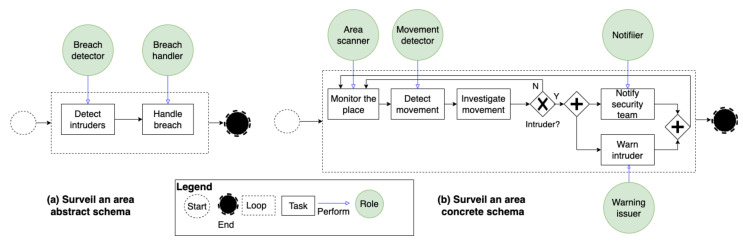
Role-based schemas that can be instantiated to surveil a factory.

MAS-based goal-driven IoT systems are dynamically formed through automatically instantiating *role-based schemas*. Instantiating schemas means dynamically assigning the roles they comprise to agents that can perform the tasks of those roles. There are two types of role-based schemas: *abstract* and *concrete* schemas. Both types comprise roles, constraints, and process models. However, the two types have differences, as described in the following. In abstract schemas, roles are assigned to application-level agents, whereas in concrete schemas, roles are assigned to dynamically discovered things-level agents. Likewise, the constraints are specified at the agents’ level in abstract schemas, while constraints are specified at the things’ level in concrete schemas. For instance, only connected and operational things can adopt roles. Constraints can also specify the number of agents that can adopt a role concurrently. Moreover, the process models in concrete schemas comprise finer grain tasks compared to the models in abstract schemas [8]. Figure 3 shows an abstract and a concrete role-based schema that can be instantiated to realize the smart surveillance system discussed in Section 4.1. After a goal-driven system is dynamically formed, it is enacted automatically, where each agent orchestrates the agents it manages to perform the tasks in the roles they adopt. Suppose an agent becomes disconnected, compromised, or unavailable; in this case, the agent that manages it adapts the system by reassigning the roles of the affected agent to one or more agents that can perform the roles’ tasks. We refer the readers to [8] for more details concerning the dynamic formation, adaptation, and enactment of MAS-based IoT systems.

#### 4.2.2. A Concrete Architecture

**Figure 4:**
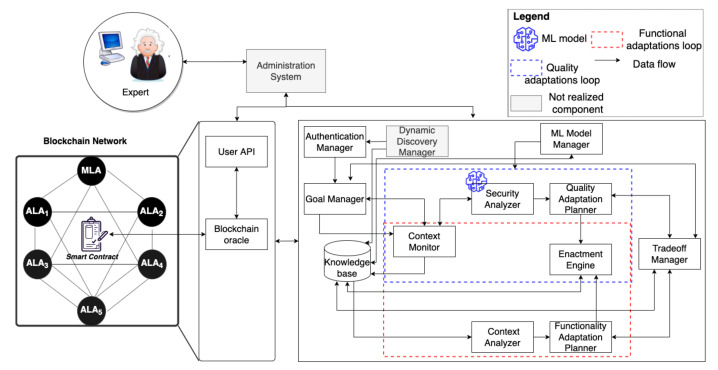
The software architecture of an agent and the blockchain network.

**Figure 5:**
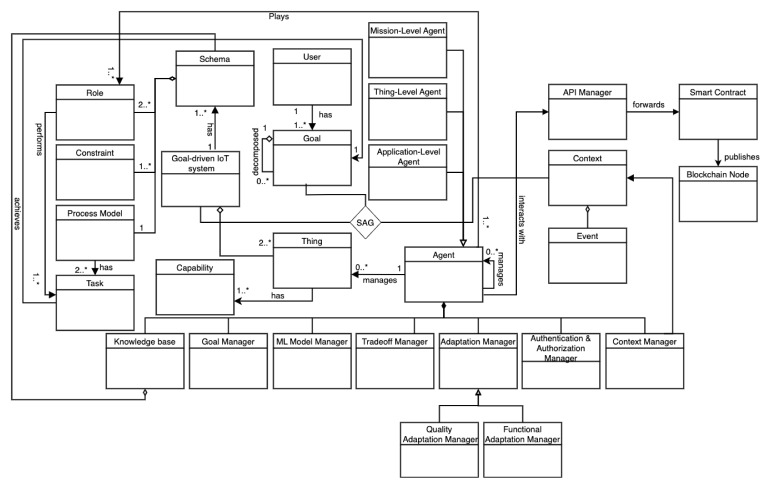
The logical view of our architecture represented in a UML class diagram.

**Figure 6:**
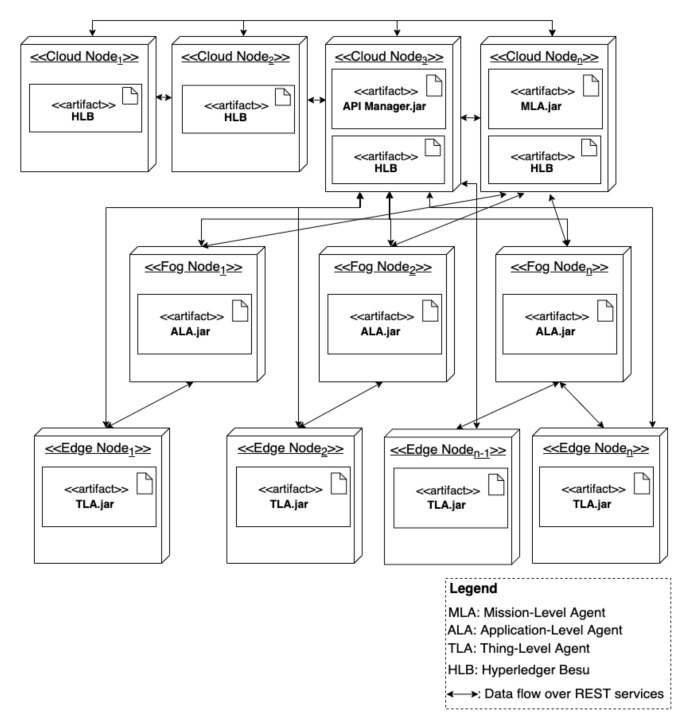
The deployment view of our approach.

To present our architectural approach, we follow the well-known 4 + 1 architectural view model [63]. Specifically, Figure 4 shows the development view represented via a components-and-connectors diagram. Figure 5 illustrates the architecture’s logical view represented via a UML class diagram. Further, Figure 6 shows the deployment view of our approach. Furthermore, Section 4.3 introduces the process view by presenting multiple processes that show the dynamic interaction among the different components in our approach. Finally, the scenarios were introduced in Section 4.1.

In principle, agents in all the layers have the same components and connectors; however, they differ in the granularity of the processes they enact and the complexity of the adaptations they perform. Specifically, each agent performs security adaptations when the agent itself or the agents it directly orchestrates in the lower layers detect security threats. For instance, in SC1, the drone that detected the attack rebooted, and the ALA that manages the drone automatically dispatched another drone to compensate for the compromised one.

The *authentication manager* is responsible for authenticating agents collaborating to realize a goal-driven and self-adaptive IoT system (**R1**). The *goal manager* is responsible for achieving an agent’s goal in a specific context by loading and instantiating the suitable role-based schema corresponding to the goal type. The achievement of agents’ goals in the specific context leads to achieving the user goal. This is represented by the ternary relation System–Achieve–Goal (SAG) presented in Figure 5. The goals of the agents and the role-based schemas they instantiate have different granularity as described in Section 2.

To enable goal-driven and self-adaptive systems to cope with changes in their environments, including security threats, two MAPE-K control loops are integrated: the functional adaptation loop and the quality adaptation loop. The functional adaptation loop is responsible for performing application-based adaptations to maintain the achievement of the agent’s goal. For instance, a drone with two cameras rotates to scan an area using the back camera after the front camera malfunctions. Another example is when an application-level agent dispatches a drone to patrol an area when the other drone that currently patrols the area is about to return to base to recharge its battery. Such adaptations have been enabled by several approaches (e.g., [8,9]). Thus, they are not addressed further in this paper.

Instead, the focus is on the quality adaptation loop, which comprises six components responsible for performing quality-based adaptations to achieve the agent’s goal with higher satisfaction. In particular, this paper focuses on security adaptations, but additional quality characteristics of goal-driven and self-adaptive IoT systems will also be addressed in future work. The *context monitor* is responsible for updating the agent’s beliefs in the knowledge base by collecting and formatting data about the agent itself and the environment in which it operates. This includes monitoring the state machine of the agent, the status of the adaptation processes, opened ports, and the agents that the agent orchestrates (in case it is an ALA or MLA). Additionally, the context monitor collects the sensors’ readings and the actuators’ status that the agent manages (in case it is a TLA).

The *security analyzer* is responsible for analyzing the data collected by the context monitor, recognizing strange behaviors, detecting security threats, and triggering events when (potential) security threats are detected (**R1**). To recognize strange behaviors, the security analyzer detects strange transitions in the agent state machine (**R3**). Examples of strange behaviors include a drone’s cameras being turned off during the patrolling state or when a drone lands suddenly after taking off and then adjusting its altitude. When a strange behavior is recognized and an attack is detected, the countermeasures are applied automatically. If no attacks are detected, the expert is notified with details about the behavior, and they decide whether to investigate it further or not. To detect attacks, the quality analyzer employs a machine learning model that an expert initially trained at the design stage (**R1**). The *ML model manager* is responsible for collaboratively and automatically training the ML model at runtime by applying federated machine learning (**R2**) [52] (see Section 4.3.2).

The *quality adaptation planner* is responsible for evaluating the events’ effects on the qualities of achieving the agent’s goal and for deciding suitable adaptation plans that can be executed to maintain those qualities if possible. Specifically, when a security threat is detected, the quality adaptation planner loads the suitable countermeasure process from the knowledge base and sends it to the enactment engine. The *trade-off manager* is responsible for handling situations where the agent aims at achieving competing concerns (**R4**). For instance, the agent’s security analysis might degrade the performance of enacting the tasks assigned to the agent and increase the energy consumption. Additionally, the security countermeasures might prevent the agent’s goal from being achieved. In such cases, the trade-off manager prioritizes the competing concerns and coordinates with the goal manager and adaptation control loops to decide the agent’s tasks. For instance, when a drone’s quality adaptation loop detects a security threat, the countermeasures might include turning off its cameras and instructing it to fly to the service center.

In a dynamic IoT environment, the blockchain plays a vital role in maintaining the data integrity and enhancing the overall trustworthiness. For this purpose, a smart contract is deployed to store the following:

(a) Details about all types of agents: These details include the agents’ identifiers, public keys, and MAC addresses that the expert provides via the *administration system* through the *user API* component. These details are used for authentication purposes as described in Section 4.3.
(b) The agents’ beliefs: The MLA’s and ALA’s beliefs are stored periodically in the blockchain network and are used when security adaptations are performed in response to security threats (see Section 4.3);
(c) Access control policy for the data stored on-chain: The policy specifies the actions that an agent is authorized to perform, including retrieving other agents’ beliefs in case of security adaptations. The *blockchain oracle* is responsible for interacting with the smart contract to store and/or retrieve data as requested by experts or agents.

The data transferred between TLAs and ALAs are encrypted using the TLAs’ private keys where only authorized ALAs can obtain the corresponding public keys from the ledger and use them for decrypting the data they receive.

Finally, the *knowledge base* (KB) is the container of agent beliefs. The agent has beliefs about the goals it can achieve, the agents or IoT devices it manages, and the enactment state machine. Additionally, the KB stores the data collected by the context monitor and the knowledge resulting from analyzing that data, the detected attack types, the triggered events and their status, and the agent’s private key.

Figure 6 illustrates the approach’s deployment topology (i.e., deployment nodes and their interconnections), which spans across the Edge–Cloud continuum. In general, the deployment of our approach and dynamically formed goal-driven IoT is complex, partly due to the following reasons. The constituents (i.e., things or services) of goal-driven IoT systems dynamically formed to achieve a goal can be different from an environment to another based on the available things. Further, goal-driven IoT systems may have different non-functional requirements (e.g., performance) based on the goals they are formed to achieve. Therefore, we use generic terms such as edge, fog, and cloud nodes in our figure. Examples of edge nodes include drones or Raspberry Pis that can run external software (i.e., TLAs). Fog nodes can, for example, be laptops that run ALAs. We refer the reader to Section 5 for the characteristics (e.g., processing capabilities) of the different nodes we used to deploy our approach and run the experiments.

**The Expert.** The expert is responsible for the following tasks:

1. Registering agents in the blockchain network via the administration system, which interacts with the network through the user API component.
2. Investigating the agents’ strange behaviors that were recognized automatically (see above) and labeling new attacks related to those behaviors (if any).
3. Periodically checking the outputs of the agents’ ML models used to detect security threats and the data collected by the agents.

Finally, the communications among the architecture components and the expert are secured using the Public Key Infrastructure (PKI).

### 4.3. Processes

This section presents the *ASSERT*processes that enable goal-driven and self-adaptive IoT systems to perform security adaptations autonomously. First, a high-level self-adaptation process within an agent is introduced. Then, detailed processes that refine the abstract process are presented to illustrate how *ASSERT*supports goal-driven and self-adaptive systems to perform security self-adaptation in response to three types of security threats. Finally, the federated learning process to collaboratively and automatically train agents’ ML models at runtime is presented.

#### 4.3.1. Security Adaptation Processes

**Figure 7:**
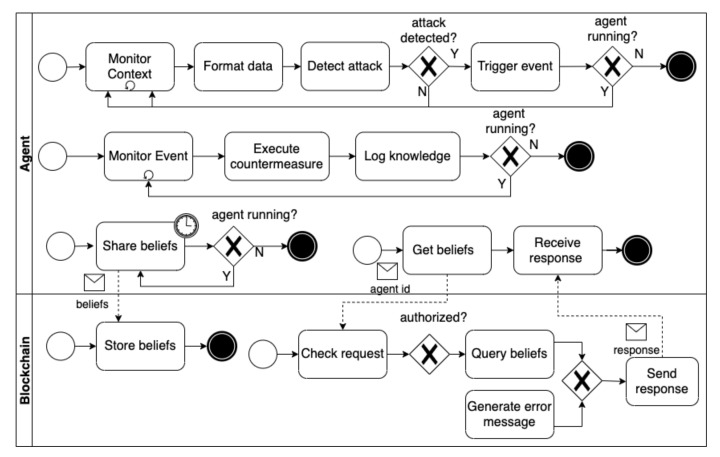
The abstract security adaptation process.

Figure 7 shows the high-level self-adaption process that enables agents to self-adapt to security threats. An agent continuously monitors and analyzes the context, including the readings of the sensors it manages (if any), the open communication ports, and the energy consumption rates. When an attack is detected, an event of the type of the attack is automatically triggered. Triggered events are automatically detected by the agent and are shared with the agent in the upper layer, which manages the agent that detected the attack. When a security threat is detected, both the agent and the managing agent self-adapt to maintain the security of the system. For instance, as described in SC1, the drone that detected the threat reboots, while the ALA managing it dispatches another drone to compensate for the compromised one.

ALAs and the MLA periodically store their timestamped beliefs in the blockchain network. If they are compromised, the agents assigned their roles can retrieve their beliefs stored in the blockchain before the attack was performed. Thanks to blockchain technology, those beliefs are trusted not to be altered by the attack.

**Figure 8:**
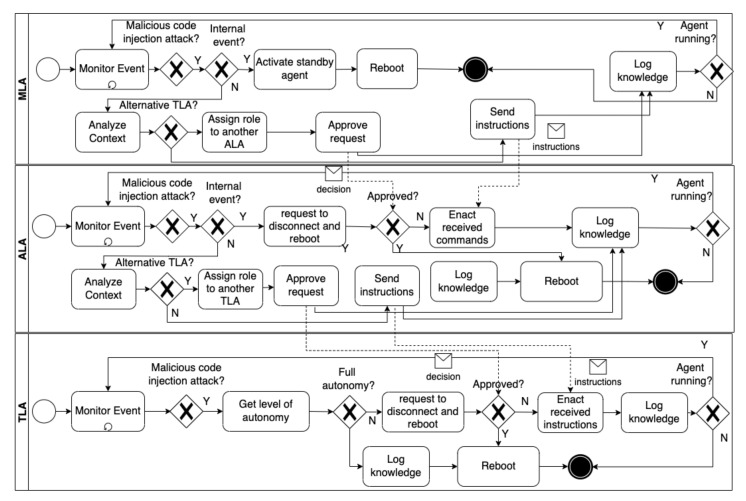
The adaptation process to a malicious code-injection attack.

**Malicious code-injection attacks.**Figure 8 shows the self-adaptation process applied when the different types of agents detect malicious code-injection attacks (For simplicity purposes, we do not show the blockchain network layer and hide some sub-processes that concern triggering events as they are described earlier in this section). TLAs can be partially or fully autonomous as most IoT devices have limited processing and storage capabilities. In the former case, a partially autonomous TLA loads the countermeasure strategy from its knowledge base (e.g., reboot), notifies the ALA managing it about the attack, and requests permission to perform the strategy. If another TLA can achieve the tasks of the compromised TLA, the ALA reassigns the role to the secure TLA and accepts the compromised TLA’s request to perform its strategy. On the other hand, the ALA might reject the request of the ALA to perform the countermeasure strategy and instead instruct it to perform another strategy.

The same process described above applies when an ALA detects an attack. However, in this case, the MLA assigns the role of the ALA to another ALA that can achieve the same goals of the compromised ALA and defines a new access role for the new ALA by sending a transaction to the smart contract in the blockchain network. Thus, the newly involved ALA retrieves the compromised ALA’s last beliefs written in the blockchain before the agent was compromised (i.e., before the threat was detected). Then, the newly involved ALA analyzes and validates the retrieved beliefs and accordingly instructs the TLAs it manages to maintain the mission achievement.

An MLA has no managing agent. Thus, depending on the criticality of the mission it operates to achieve, a standby backup MLA might be needed. The standby MLA is activated automatically when the active MLA detects an attack, while the compromised MLA reboots to mitigate the attack.

**Figure 9:**
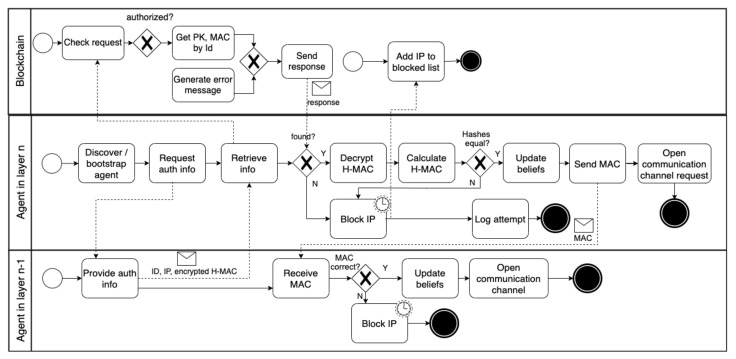
The dynamic authentication process (mitigates fake node attacks).

**Fake node attacks.** Agents forming goal-driven and self-adaptive IoT systems should be authenticated to prevent fake node attacks. Following our hierarchical architecture, mutual authentications should be conducted between the MLA and all the ALAs and between the ALAs and the TLAs they manage. As already mentioned in Section 4.2.2, the identifiers, MAC addresses, and public keys of all the agents are stored by the expert in the blockchain network. Figure 9 shows the dynamic authentication process. For instance, when a TLA is discovered in the spatial boundaries within an ALA’s responsibility, the ALA requests the TLA to provide its authentication information (i.e., ID, IP, and encrypted H-MAC). Then, the ALA requests the blockchain to provide the TLA’s public key and MAC address. The blockchain responds with the requested information if the ALA is authenticated and authorized. Otherwise, an error message (e.g., 401 unauthorized error) is returned.

Thereafter, the ALA decrypts the H-MAC using the retrieved public key, calculates the H-MAC, and compares the result with the H-MAC shared by the TLA. If both H-MAC addresses are equal, the ALA updates its beliefs about the TLA and adds it to the list of agents it manages. Then, the ALA forwards the MAC address that was retrieved from the blockchain to the TLA. If the MAC address is correct, the TLA updates its beliefs and specifies the ALA as its managing agent. When the TLA (e.g., a drone) moves to different boundaries, the same process is repeated with the ALA responsible for managing the TLAs within those boundaries. At the same time, the dynamic discovery service in the former ALA manager updates the ALA beliefs, affirming that the TLA is no longer managed by it. Finally, an MLA is authenticated offline by the expert. The MLA and the ALAs mutually authenticate in the same way described above. However, the authentication processes can be conducted during the agents’ bootstrapping phase.

**Figure 10:**
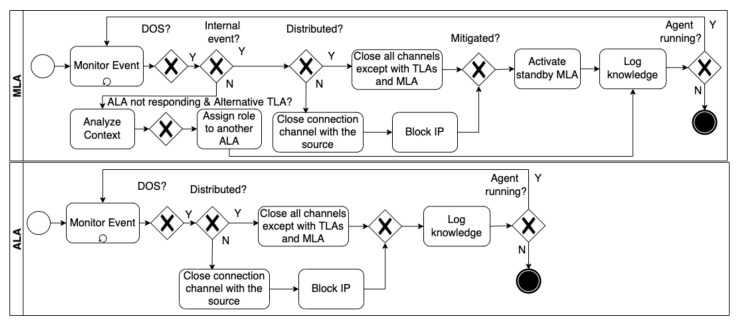
The adaptation process to a denial-of-service attack.

**Denial-of-service attacks.**Figure 10 shows the adaptation process to denial-of-service (DoS) attacks targeting ALAs and an MLA. When an ALA detects a DoS attack, it notifies the MLA, as described in the abstract self-adaptation process. If the detected attack originates from a single source, the ALA closes the connection channel with the source and blocks its IP address. If the attack is distributed, the ALA closes all the communication channels except with the TLAs it manages and with the MLA. Meanwhile, the MLA keeps monitoring the status of the ALA. If the latter does not respond, the MLA adapts the system by assigning the role of the ALA to another ALA that can achieve the same goal. The same process applies when DoS attacks target TLAs and the MLA, but the standby MLA is activated automatically when the MLA goes down.

#### 4.3.2. Federated Learning Process

**Figure 11:**
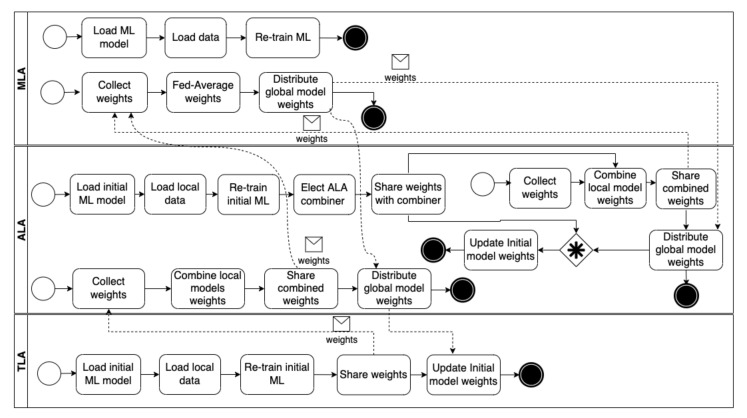
The federated learning process.

Figure 11 shows the federated learning process that enables agents to train their ML models to detect security threats collaboratively. First, a TLA loads the initial ML model, trained offline by an expert, from its knowledge base. Then, it loads local data that have been collected while the agent runs and uses the data to re-train the initial ML model. Thereafter, the TLA shares the weights of the re-trained model with the ALA managing it. Next, the ALA combines the weights shared with the models of the same features and shares the combined weights with the MLA. Later, the MLA calculates the models’ average in the same category and distributes the average weights back to the TLAs via the ALAs managing them.

After the ALAs re-train their models locally, the ALAs that have models with the same features and operate in the same network elect an ALA to combine the resulting weights. Then, elected ALAs from different networks share the weights with the MLA, which calculates the average weights of the models, and distributes the average weights back to the ALAs via the elected ALAs. If an elected ALA is not available, the MLA shares the averaged weights with the ALA currently playing its role. Finally, the MLA incrementally trains its ML model by re-training it, using the data it collected while managing the self-adaptive and goal-driven IoT systems.

## 5. Validation

To validate the feasibility of our approach and evaluate its performance and scalability, a prototype that implements both the *ASSERT*architecture and the processes was developed and used to run the following experiments:

1. **Experiment 1**: Evaluate the performance of agents deployed on Edge nodes with different capabilities when detecting security threats automatically.
2. **Experiment 2**: Evaluate the accuracy of the ML models collaboratively trained to detect security threats through applying the federated learning process presented in Section 4.3.2 and evaluate the scalability of the process. This experiment was run on the Swedish National Infrastructure for Computing Science Cloud (SNIC) [64].
3. **Experiment 3**: Evaluate the performance and scalability of *ASSERT*when adapting to security threats. This experiment was run on the Amazon AWS Cloud platform (https://aws.amazon.com/ accessed on 30 August 2022).

**Experiment 1.** In this experiment, the NSL-KDD dataset was used to train agents’ ML models to detect security threats automatically. The dataset (https://www.unb.ca/cic/datasets/nsl.html accessed on 30 August 2022) comprises more than 125,973 training samples, where 47% of the samples represent security threats to IoT systems. The testing data comprises 22,544 samples, of which 57% represent security threats to IoT systems. Several ML algorithms have been compared in centralized settings in terms of accuracy and performance using five-fold cross-validation to choose an algorithm for detecting security threats. The results presented in Table 1 revealed that the four algorithms that achieved the highest classification performance with small differences were long short-term memory, gradient boosting, light gradient boosting, and decision tree, respectively. In the developed prototype, the LSTM algorithm was used for enabling agents to detect security threats.

**Figure 12:**
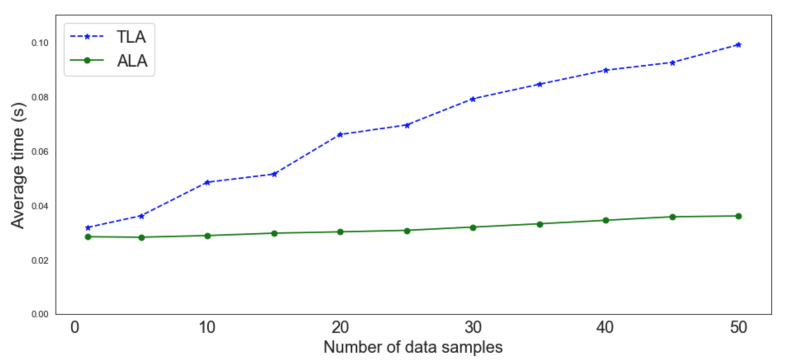
ML execution time on an ALA and a TLA.

To evaluate the performance of the ML models to detect security threats, the model was deployed in two agents: (1) an ALA running on a computer having 16 GB RAM and a 2.2 GHz eight-core CPU; and (2) a TLA running on a Raspberry Pi 4 (https://www.raspberrypi.com/products/raspberry-pi-4-model-b/ accessed on 30 August 2022) having 4 GBs of RAM and a 1.5 GHz quad-core CPU. Figure 12 shows the execution time on both agents. Different sizes of data samples were considered as inputs to the models. The results show that detecting security threats on both agents using ML models is a responsive and not-resource-demanding process.

**Experiment 2.** To collaboratively train the ML models, the FEDn platform (https://github.com/scaleoutsystems/fedn accessed on 30 August 2022) was used to realize our federated learning process. The FEDn platform was chosen because it is modular, model agnostic, and supportive of hierarchical federated learning, making it aligned with the hierarchical architecture of *ASSERT*. The FEDn was deployed at the Swedish National Infrastructure for Computing Science Cloud (SNIC). The ALAs and the MLA were deployed on virtual machines having 16 GB RAM and 2.2 GHz eight-core CPUs, whereas the TLAs were deployed on virtual machines that simulate a Raspberry Pi 4 with 4 GBs of RAM and a 1.5 GHz quad-core CPU.

**Figure 13:**
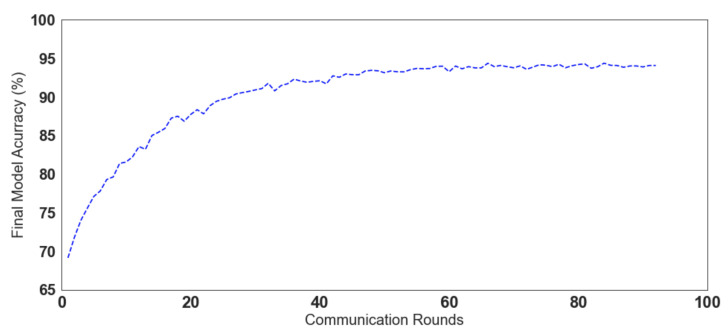
Collaborative training accuracy using the FEDn framework.

Figure 13 shows the accuracy convergence of the LSTM model when trained collaboratively following the *ASSERT*federated learning process presented in Section 4.3.2. First, the seed model was initiated with random weights and distributed to the involved agents. Then, the agents trained the models locally using their private data. Accordingly, the global model’s weights were updated by averaging the weights resulting from the local training rounds. As the figure indicates, the final global model’s accuracy converges better with more training rounds.

**Figure 14:**
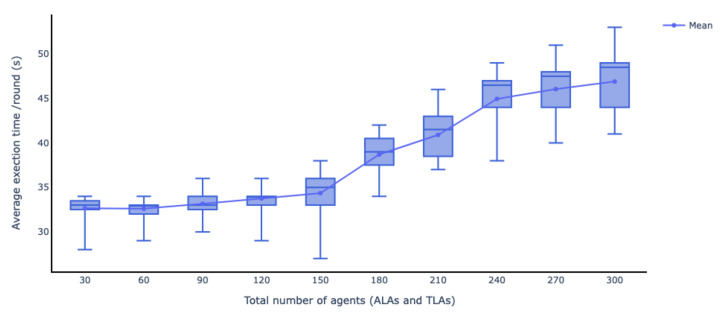
The scalability of the federated learning process using the FEDn framework.

Moreover, to evaluate the scalability of the federated learning process, another experiment was conducted to measure the average time it takes an increasing number of agents to perform complete training rounds. Each ALA is considered to orchestrate five TLAs, and one MLA orchestrates all ALAs. As can be seen in Figure 14, when there are 50 ALAs and 250 TLAs, the average time of completing 15 training rounds takes approximately 46 sec.

**Experiment 3.** To evaluate the performance and scalability of *ASSERT*when self-adapting to security threats, a blockchain network of four nodes was implemented on the Amazon AWS Cloud platform. For each node, a virtual machine of the type t3.2xlarge that had 8 vCPUs and 32 GBs of RAM was used. The Hyperledger Besu (https://www.hyperledger.org/use/besu accessed on 30 August 2022) was used to run the blockchain protocol on each node. The smart contracts were executed in the Hyperledger Besu private platform to operate the business operations of the agents. The smart contracts were developed using solidity programming language, and they are deployed and run in the Remix IDE environment (https://remix.ethereum.org/ accessed on 30 August 2022). Further, another virtual machine was created to host the backend server that was developed using Nodejs (https://nodejs.org/en/ accessed on 30 August 2022) to expose the APIs through which agents could interact with the blockchain to read or write data.

**Figure 15:**
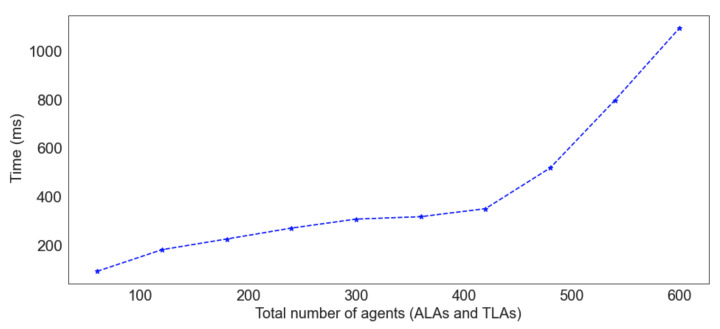
Mutual authentication and self-adaptations performed to mitigate fake node attacks.

To evaluate the scalability and performance of ASSERT, the process that mitigates fake node attacks (see Section 4.3) was implemented. This type of threat was chosen because its mitigation process involves more complex sub-processes than the two other processes—including reading and writing data from and to the blockchain, encrypting and decrypting data using public and private keys, and updating the agents’ beliefs. Furthermore, it is considered that each ALA mutually authenticates five TLAs and that 20% of the TLAs are fake nodes. Additionally, to simulate dynamic environments, 15% of the TLAs were considered to be mobile, so the ALAs that manage them have changed at runtime. As can be noted in Figure 15, *ASSERT*scales and performs well when authenticating agents and mitigating fake node attacks.

## 6. Discussion

Engineering secure IoT systems is a key element in adopting those systems in our daily life. The large number and varying characteristics of IoT devices (concerning their processing and storage capabilities and energy sources), in addition to the dynamicity and uncertainty of IoT environments, demand novel approaches to secure IoT systems. Toward achieving this goal, we proposed ASSERT, an approach that enables goal-driven IoT systems to detect security threats and self-adapt to mitigate those attacks. The results of the experiments conducted to validate the approach’s feasibility show that it can perform and scale well when the approach is deployed across the Edge–Cloud continuum. However, our approach faces the following challenges that we plan to address in future work. The first challenge concerns the performance of manipulating data on constrained blockchain nodes deployed on the edge of the network. Blockchain technology supports recording information about agents securely in a distributed and trusted manner. It has also been used for access control purposes to decide the agents’ access permissions and under which conditions. Further, blockchain is used by standby agents to read their ancestor’s beliefs. The experiments’ results showed that manipulating data in blockchain is accomplished in a reasonable and acceptable time when the blockchain network is deployed in distributed settings at the Cloud. However, we envision that the time needed to manipulate data will increase when the blockchain network is deployed in more constrained edge nodes in fully decentralized settings or when the number of participating agents increases. As future work, the usage of off-chain storage (e.g., on the InterPlanetary File System (https://ipfs.io/ accessed on 30 August 2022) will be investigated to address performance issues related to manipulating big data.

The second challenge concerns the accuracy for detecting security threats. AI enables the detection of security threats using ML models and the autonomous adaptation to those threats using the notion of agents. In our prototype, ML models could detect threats with high accuracy. However, in principle, those models can detect threats with low accuracy or even fail to detect those threats, for example, due to the quality of the training data. In the former case, the ML models become an additional source of uncertainty and thus increase the complexity of the adaptation processes. For instance, the ML models can report false positive attacks, which trigger unneeded adaptation processes that would affect the functionalities of IoT systems and consume their resources. Moreover, ML models are not able to detect zero-day attacks. Toward addressing these challenges, our approach involves experts in the loop and integrates federated learning to enable the collaborative and incremental learning of ML models to detect security threats. The third challenge concerns performing security adaptations re-actively. Our experiments show that the approach performs and scales well in detecting security threats and adapting to those threats in dynamic environments. However, our approach enables re-active security as it requires the detection of security threats to trigger the adaptation processes. Our approach will be evolved to provide predictive security adaptations, where agents can predict threats before they manifest themselves. This can be achieved by logging the events and the agents’ properties and states that precede detecting threats and using that knowledge to predict those threats in the future.

Finally, cases where adversarial attacks are performed on ML models were not investigated, and it was assumed dynamic discovery services were available to monitor and track agents. These issues will be addressed in future work.

## 7. Related Work

Several approaches have been developed to engineer goal-driven and/or self-adaptive IoT systems. A service-oriented approach presented by [9] supports users to achieve their goals by enabling the dynamic composition of semantically annotated web services that wrap things’ capabilities, considering high-level security policies. For instance, a user could specify a security requirement to have communications over HTTPs. The approach also supports the dynamically formed goal-driven IoT systems to self-adapt apropos the availability of services or changes in the security policies. Another approach by [10] exploits logic programming to enable the dynamic composition of web services. Further, Chen et al. [65] presents GoCoMo, a goal-driven and self-organizing model that exploits heuristic and AI planning to enable the decentralized automated composition and adaptation of services in mobile and pervasive environments. An agent-based approach proposed by [7] enables the dynamic formation of goal-driven IoT mashups to support users to achieve their goals. Additionally, De Sanctis et al. [66] introduce a service-based approach for enabling the dynamic formation of goal-driven IoT systems considering some quality aspects of the available things (e.g., response time). Finally, a number of approaches for realizing goal-driven and self-adaptive IoT systems are presented in [17]. As shown in Table 2, unlike *ASSERT*, the existing approaches proposed to engineer goal-driven and self-adaptive IoT systems do not enable the automated detection of security threats and the autonomous adaptations to mitigate them.

Unlike the traditional monolithic systems, IoT deployment architectures are vast models, requiring several interacting agents (i.e., multi-agent systems) in the decision process. However, such intelligent distributed systems impose privacy and security concerns. Revealing metadata or services securely to other agents and exchanging data is a leading issue. In this regard, blockchain technology aids in creating a privacy-preserving and transparent communication system—using a peer-to-peer network with a cryptographic hash and timestamp. Many studies have examined adopting blockchain network services to multi-agent systems. In [68], the authors propose an adaptive tit-3-for-tat strategy that encourages collaborating for three consecutive rounds of play to improve the trusted credibility. However, their study does not consider a common blockchain engine that tracks the network with a quality analyzer representing the multi-agent system’s functional adaptation loop. Integrating multi-agent systems and blockchain technology was attempted by Yang Fan et al. [69] to analyze social networks. Pertinent research by [70] focuses on leveraging blockchain to realize an intelligent IoT architecture and highlights distinct parameters that allow the convergence of blockchain, AI, and IoT. In addition, the performance evaluation emanating from this proposition explicitly points to security as a key parameter needed in engineering blockchain-based IoT systems.

In [71], the authors use a private blockchain as a measure of ensuring secure communication and to encounter several attacks, such as Man-in-the-Middle (MiTM), impersonation, and insider attacks, that can target the AI-enabled Internet of Drones (IoD). A DAG-structured blockchain and a credit-based PoW mechanism are used in [72] to balance the trade off between efficiency and security in an IoT system. Further, integrating AI models over the network’s edge was proposed for IoT sensors forensic architecture using learning-enabled systems [73].

Few approaches have exploited ML techniques to detect and self-adapt to security threats. Most existing approaches adopt centralized architectures and focus mainly on soft-security requirements such as trust to achieve the minimum level of security [18]. In [74], the authors consider trust as an emergent solution for the soft security of self-adaptive systems by examining various trust evaluation approaches. Their results show that none of the studied approaches were suitable as they did not properly tackle the characteristics of self-adaptive systems. In [75], trust is also identified as one of the important criteria to achieve the minimum level of security in the open and distributed environment. The authors proposed trust-aware goal modeling for cooperative self-adaptive systems. Their model aimed to consider situations where trust is required at the requirement engineering process, allowing system engineers to consider and represent trust as an attribute at the very early stage of system development.

Further, Jahan et al. [76] introduce an approach that exploits two control feedback loops to manage runtime adaptations in response to evolving functional and security requirements. Additionally, the authors present an interaction protocol to coordinate the functional-driven and security-driven adaptations. Similarly, authors in [77] propose an adaptive decentralized approach for developing trustworthy information-flow control by employing feedback loops. Finally, in [78], the authors extend the traditional MAPE-K to perform dynamic architectural reconfigurations. Their work utilizes reinforcement learning to select the optimal adaptation policy by monitoring the quality-of-service parameters and running feedback techniques to evaluate and adapt the selected decisions.

Several approaches have exploited blockchain to engineer secure and trustworthy systems [79]. Wang et al. [80] focus on security risks associated with data storage in cyber–physical systems and propose a blockchain-based approach. The authors highlight that traditional Merkle hash trees for data storage cannot add or delete elements or prove the (non-)membership of certain elements. Therefore, they use a combination of accumulator and Merkle hash tree to provide those features in addition to assuring security. *ASSERT*instead follows an agent-based goal-driven approach that could use self-adaptation to tackle security risks and use smart contracts to authenticate agents and secure data sharing. In [81], the authors analyze the challenges concerning secure data storage in sensor networks. Further, they propose a blockchain-based approach to enforce data security in sensor networks. Their approach focuses on building a cryptographic unbounded accumulator as a step toward data security, whereas *ASSERT*focuses more on engineering secure and self-adaptive IoT systems by exploiting multiple technologies, including blockchain. Xu et al. [82] propose a decentralized blockchain-based authentication protocol for the Internet of Vehicles. The approach exploits blockchain to create a network of trusted authorities’ nodes that can not only achieve cross-nodes authentication of vehicles but also improve authentication efficiency. Adaptations can also be performed at the level of smart contracts in blockchain. Górski proposes a pattern to enable the runtime reconfiguration of a smart contract to adjust for various transaction types. Additionally, the pattern supports the re-use of verification rules between different smart contracts’ configurations [83]. In our approach, at this stage of the work, we focus on the adaptations to mitigate security threats at the level of agents, and we use blockchain as a trustworthy repository of agents’ beliefs to enable trustworthy adaptations.

To summarize, to the best of our knowledge, *ASSERT*is the first approach that takes the software engineering perspective to enable automatically formed goal-driven IoT systems to self-adapt to security threats in dynamic and uncertain environments. Specifically, *ASSERT*supports these systems to perform trustworthy, autonomous, and distributed security adaptations and learn about security threats in their environments collaboratively—empowered with the agents, machine learning, control loops, and blockchain technologies.

## 8. Conclusions and Future Work

This paper proposes an architectural approach that enables autonomous, trustworthy, and secure self-adaptations of goal-driven IoT systems called ASSERT. ASSERT architecture capitalizes on the recent advancements in federated learning and blockchain technologies, mitigating the associated IoT security threats. The approach comprises an architecture, security self-adaptation processes, and a prototype that was developed and used to run experiments to validate the approach’s feasibility. The experiments’ results show that the approach scales and performs well when detecting security threats, performing adaptations to mitigate them, and when collaboratively training ML models to improve their accuracy in detecting those threats.

In future work, we plan to evolve the approach to address adversarial attacks on ML models used to detect security threats, consider additional types of security threats and develop processes to mitigate them, and develop dynamic discovery and tracking services. Moreover, we plan to consider additional non-functional requirements, such as availability, reliability, and robustness. Further, we plan to propose methods to predict security attacks and to enable the automated registration of agents at runtime. Furthermore, we plan to extend the developed prototype and conduct a more extensive evaluation. Finally, we plan to investigate how self-adaptations can be performed in a fully decentralized manner, where agents can be resource-constrained and edge-based blockchain nodes.

## Figures and Tables

**Table 1:** The classification performance of the ML algorithms.

Algorithm	Accuracy	Cohen’s Kappa	F1 Score	MatthewsCoff
LSTM	88.44%	77.02%	0.89	0.78
Gradient Boosting	85.89%	72.11%	0.86	0.74
Light Gradient Boosting	85.31%	71.05%	0.85	0.73
Decision Tree	85.23%	70.95%	0.85	0.73
Support Vector Machine	85.16%	70.72%	0.85	0.73
eXtreme Gradient Boosting	84.94%	70.35%	0.85	0.73
Bagging Classifier	84.29%	69.13%	0.84	0.72
Random Forest	83.49%	67.64%	0.83	0.70
K-nearest Neighbor	82.04%	64.91%	0.82	0.68
ExtraTree	81.89%	64.64%	0.82	0.68
Gaussian Naive Bayes	80.39%	61.32%	0.80	0.63

**Table 2:** A comparison with existing approaches proposed to engineer goal-driven and self-adaptive (IoT) systems.

Approach	Architecture	Dynamic Formation	Security Adaptations	Trustworthy Adaptations	Collaborative Learning
[6,67]	Centralized	**✓**	**✗**	**✗**	**✗**
[9]	Not presented	**✓**	partially	**✗**	**✗**
[10,65]	Not presented	**✓**	**✗**	**✗**	**✗**
[8,66]	Distributed	**✓**	**✗**	**✗**	**✗**
[7]	Decentralized	**✓**	**✗**	**✗**	**✗**
ASSERT	Distributed	**✓**	**✓**	**✓**	**✓**
